# Maternal efficacy and sedentary behavior rules predict child obesity resilience

**DOI:** 10.1186/s40608-015-0057-1

**Published:** 2015-06-20

**Authors:** David Crawford, Kylie Ball, Verity Cleland, Lukar Thornton, Gavin Abbott, Sarah A McNaughton, Karen J Campbell, Johannes Brug, Jo Salmon, Anna Timperio

**Affiliations:** Centre for Physical Activity and Nutrition Research, School of Exercise and Nutrition Sciences, Deakin University, 221 Burwood Highway, Burwood, 3125 Australia; Menzies Research Institute, University of Tasmania, Hobart, Australia; EMGO+ Research Institute, VU University Medical Center, Amsterdam, Netherlands

**Keywords:** Familial environment, Neighborhood environment, Resilience, Socioeconomic disadvantage, Weight gain

## Abstract

**Background:**

To identify longitudinal individual, social and environmental predictors of adiposity (BMI z-score), and of resilience to unhealthy weight gain, in healthy weight children and adolescents.

**Methods:**

Two hundred healthy weight children aged 5–12 years at baseline and their parents living in socio-economically disadvantaged neighborhoods were surveyed at baseline and three years later. Children’s height and weight were objectively measured, parents completed a detailed questionnaire that examined the home, social and neighborhood environments, and objective measures of the neighborhood environment were assessed using geographic information system data. Children classified as healthy weight at baseline who had small or medium increases in their BMI z-score between baseline and three year follow up (those in the bottom and middle tertiles) were categorized as ‘resilient to unhealthy weight gain’. Where applicable, fully adjusted multivariable regression models were employed to determine baseline intrapersonal, social and environmental predictors of child BMI z-scores at follow-up, and resilience to unhealthy weight gain at follow-up.

**Results:**

Maternal efficacy for preventing their child from engaging in sedentary behaviors (B = −0.03, 95 % CI: −0.06, 0.00) was associated with lower child BMI z-score at follow up. Rules to limit sedentary behaviors (OR = 1.14, 95 % CI: 1.03, 1.25) was a predictor of being resilient to unhealthy weight gain.

**Conclusion:**

The findings suggest that strategies to support parents to limit their children’s sedentary behavior may be important in preventing unhealthy weight gain in socioeconomically disadvantaged communities.

## Background

Childhood obesity has increased dramatically in recent years [1]. This is of concern because obesity in childhood impacts health in both the short and long term [2]. In addition, there is evidence that obesity in childhood is an independent risk factor for adult obesity [2]. As a consequence, promoting energy balance by supporting healthy eating and physical activity habits during childhood is critically important. In order to develop interventions aimed at preventing childhood obesity there is a need to better understand the underlying drivers of eating and physical activity behaviors. This is particularly the case for those living in socioeconomically disadvantaged circumstances, given that obesity and its predictor behaviors are socioeconomically patterned, with those of lower socioeconomic position being more likely to be overweight or obese [3], to have lower levels of physical activity [4], higher levels of sedentary behavior [5], and to demonstrate poorer dietary behaviors [6].

Previous studies have attempted to investigate the drivers of obesity by establishing the correlates of obesity, adjusting for socioeconomic effects. An alternative, but less-utilized approach to understanding the mechanisms underlying the higher rates of obesity amongst those who are socioeconomically disadvantaged involves investigation of the characteristics and circumstances of those who manage to maintain a healthy weight, despite being exposed to circumstances (such as socioeconomic disadvantage or obesogenic environments) that increase obesity risk. As we have argued previously [7], not everyone experiencing socioeconomic disadvantage is overweight or obese, or gaining weight. Some socioeconomically disadvantaged individuals manage to eat well, remain physically active, and maintain a healthy weight. We have suggested that this may represent a form of ‘resilience’. Investigating the characteristics that support such resilience represents a novel approach to understanding and preventing obesity in high-risk groups. For example, in a cross-sectional study of women living in disadvantaged neighborhoods, we identified behavioral characteristics, including greater leisure-time physical activity and lower intakes of fast foods and soft drinks, associated with resilience to obesity; that study also found that cognitive, social and neighborhood characteristics all contributed to explaining variation in BMI in the hypothesized directions [8]. However, there has been relatively little research that has examined resilience in relation to obesity and studies among children are currently lacking.

Traditionally, research aimed at identifying influences on childhood obesity has focused on characteristics within and between individuals, such as attitudes, motivations, social norms, and the family environment [9]. That research has shown, for example, that parenting practices [10], parental modeling of behavior [11] and aspects of the home environment such as having a television in a child’s bedroom [12] are associated with obesity in children. In recent years, characteristics of the neighborhood environment have also come to be recognized as playing a key role. A recent review concluded that a range of neighborhood features are associated with childhood obesity, including walkability of the neighborhood, mixed land use, the presence of accessible destinations, and access to convenience stores, supermarkets and farmer’s markets [13]. However, there remain few prospective studies that have empirically assessed environmental influences on obesity. Furthermore, although there is growing recognition of the need to understand the multiple levels of influence on obesity in order to identify mechanisms that explain obesity risk [14], there has been a dearth of longitudinal research that has concurrently examined a comprehensive range of contextual influences (individual, social and environmental) on obesity, and none on resilience to obesity, among children.

We previously reported the findings of cross-sectional multivariable analyses which showed that among 5–12 year old children living in socioeconomically disadvantaged neighborhoods, a number of potentially modifiable features of the home, but not the social or neighborhood environment were associated with children’s BMI z-scores [15]. Using data from the 3-year follow up, the aims of the current study were to identify longitudinal individual, social and environmental predictors of adiposity (zBMI), and of resilience to unhealthy weight gain, amongst healthy weight children from socioeconomically disadvantaged neighborhoods.

## Methods

### Participants

Ethical approval for this study was granted by the Deakin University Human Research Ethics Committee, the Catholic Education Office and the Victorian Department of Education and Early Child Development. Initial (T1) data were collected during 2007–8, and follow-up (T2) data were collected during 2010–11. Using the Australian Bureau of Statistics’ 2001 Socio-Economic Index for Areas (SEIFA), an indicator of area-level disadvantage based on census data [16], areas within the bottom third of the SEIFA distribution for Victoria, Australia, comprised the sampling frame. Forty urban and 40 rural socioeconomically disadvantaged areas were randomly selected.

One-hundred and fifty women aged 18–45 years from each of the 80 areas were randomly identified from the Australian electoral roll (*n* = 11,940; in some areas where there were fewer than 150 eligible women, all eligible women were sampled); 4,934 women (41 %) provided informed consent and responded to a postal invitation to complete a questionnaire. Data were excluded for 585 respondents where the respondent had moved from the sampled area prior to completing the survey, where the person who completed the survey was not the intended participant, where respondents withdrew their data after completing the survey, or where respondents were outside the selected age range. Of the 4,349 eligible women, those with a 5–12 year old child (*n* = 1,457) were also invited to complete a survey about their child (selected using the next-birthday method), with 771 agreeing to do so. Child surveys were received from 613 mothers at T1. These participants were invited to be part of a follow-up cohort. Of the women with children who had participated at T1, 360 provided data at T2, on average 3.0 (SD = 0.1) years later. Participants missing data on any of the study variables (*n* = 98) were excluded from analyses. Of the remaining 262 children, 200 were classified as being in the healthy weight range at baseline and were included in the analyses (described below). Excluded participants (*n* = 413) did not differ from the retained sample with regard to maternal age; however, children in the excluded group were older (M = 9.6 years, SD = 2.1 vs M = 9.0, SD = 2.2), had higher BMI z-scores at T1 (M = 0.8, SD = 0.9 vs M = −0.1, SD = 0.7) and T2 (M = 1.2, SD = 0.8 vs M = 0.0, SD = 0.8), and were less likely to have tertiary level maternal education (22.1 % vs 34.0 %) than those included in the study sample.

### Measures

#### Demographic information

Demographic information for each child-mother pair was collected at T1. The age of each child at the time their height and weight were measured was recorded, along with their sex. Maternal age and highest level of education were self-reported by mothers. Responses were collapsed into three categories of maternal education: low (“no formal education” or “year 10 or equivalent”), medium (“year 12 or equivalent”, “trade/apprenticeship”, or “certificate/diploma”), and high (“university degree” or “higher university degree”).

#### Adiposity

Research staff attended each child’s school or home at both T1 and T2 and measured height using a portable stadiometer (Wedderburn Seca) and weight using digital scales (Wedderburn Tanita model no. TIBC351). Body mass index (BMI) was calculated for each child by dividing weight by height squared (kg/m^2^). At both time points (T1 and T2), weight status (underweight, healthy weight, overweight, or obese) was determined using Cole’s cut points [17] and age- and sex-adjusted BMI z-scores (zBMI) were calculated for each child based on the CDC reference population [18]. A zBMI change score was calculated for each child by subtracting their initial (T1) zBMI from their T2 zBMI.

#### Resilience to unhealthy weight gain

**Fig. 1:**
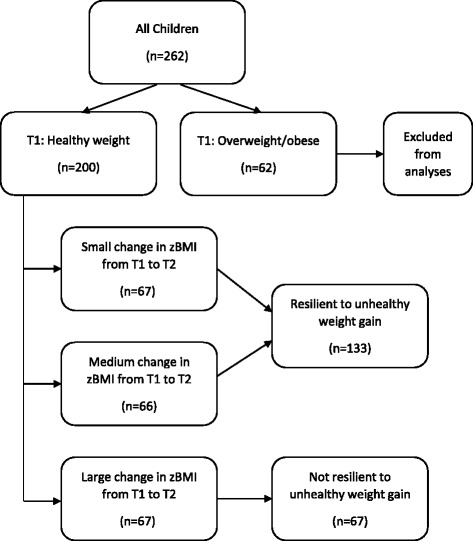
Definition of resilience to unhealthy weight gain

To classify resilience to unhealthy weight gain (Fig. 1), we first restricted our sample to children who were classified as healthy weight at T1, based on Cole’s cut points (*n* = 200) [17]. zBMI change scores were then classified into three categories (low, medium, high zBMI change score) based on thirds of the distribution (i.e. tertiles). Children who had the greatest increases in their zBMI (those in the highest third) were categorized as ‘not resilient to unhealthy weight gain’, while children who had small or medium increases in their zBMI (those in the bottom and middle third) were categorized as ‘resilient to unhealthy weight gain’.

#### Home environment

Measures of the home environment were reported by mothers. These have been described in Table 1 and included measures of: maternal efficacy for the child doing physical activity; maternal efficacy for preventing the child engaging in screen-based behaviors; maternal efficacy for the child eating healthily; parental support for physical activity; maternal perception of the importance of doing physical activity as a family; views on the use of food as a reward; views on the use of screen-based behavior as a reward; having rules to limit screen-based behaviors; mother’s feelings and beliefs about food enjoyment; home access to physical activity equipment; home access to opportunities for screen-based behaviors; and the child having access to a television in their bedroom.

**Table 1:** Home, social and neighborhood environment factors reported by mothers at baseline (T1)

Measures	Survey items	Scale and range	Descriptives/scale reliability
*Home environment*			
Maternal efficacy for child doing physical activity (10 items)	I think my child could be physically active: ‘no matter how busy his/her day is, ‘no matter how tired he/she may feel’, ‘even if it is hot or cold outside’, ‘even if he/she has a lot of homework’, ‘after school even if he/she could watch TV or play video games instead’, ‘even if he/she had to stay at home’, ‘even when he/she would rather be doing something else’, ‘even if his/her friends didn’t want him/her to’, ‘after school even if his/her friends wanted him/her to do something else’, ‘at least 3 times a week for the next 2 weeks’ [25]	4 point: 1 = not at all confident, 4 = very confident; range: 10-40	Mean: 31.1 ± 4.7; Cronbach’s alpha (α) = 0.90
			Test-retest: κ = 0.46-0.64
Maternal self-efficacy for preventing child from engaging in sedentary behaviors (3 items)	How confident are you that you could do the following over the next year? ‘Say no to my child’s demands to watch TV/videos/DVD’, ‘Say no to my child’s requests to play on the computer’, ‘Get my child to do something physically active, like dancing, skipping, playing outside, when they want to play on the computer or watch TV’	5 point: 1 = not at all confident, 5 = extremely confident; range: 3-15	Mean: 12.0 ± 2.7; α = 0.82
			Test-retest: κ = 0.46-0.55
Maternal self-efficacy for child eating healthily (6 items)	How confident are you that you could do the following over the next year? ‘Get my child to eat enough fruit (this does not include fruit juice)’, ‘Get my child to eat enough vegetables (this does not include potato or potato chips)’, ‘Get my child to drink plain water (with no flavors added)’, ‘Say no to my child’s requests for soft-drinks, cordials or other sweetened drinks’, ‘Say no to my child’s requests for potato chips/Twisties/Cheezels or similar foods’, ‘Say no to my child’s requests for sweet snacks, confectionary, lollies or ice-cream’	5 point: 1 = not at all confident, 5 = extremely confident; range: 6-30	Mean: 23.8 ± 4.4; α = 0.79
			Test-retest: κ = 0.47-0.61
Parental support for physical activity (4 items)	How often do the following people provide support for your child’s participation in physical activity? (e.g. take him/her to training, provide money for participation, buy sports clothing/equipment): ‘You’, ‘Child’s co-carer’ (these 2 scores were subsequently summed to indicate parental support). How often do each of the following people praise your child for participating in physical activity? (e.g. say positive things to him/her, seem happy that he/she does it) : ‘You’, ‘Child’s co-carer’ (these 2 scores were subsequently summed to indicate parental praise; parental support and parental praise scales were then summed to provide an indicator of overall parental support/praise, termed ‘parental support for physical activity’) [26]	1 = don’t know/doesn’t apply, 2 = never, 3 = less than once per week, 4 = 1-2 times per week, 5 = 3-4 times per week, 6 = 5-6 times per week, 7 = daily (subsequently recoded into times/week scores); range: 0-28	Mean: 12.9 ± 6.5; α = 0.74
			Test-retest: ICC = 0.81-0.90 [27]
Importance of doing physical activity as a family (1 item)	How important is it (to you) that the family does sport or other physical activity together (e.g. goes for walks)?	1 = not really important, 2 = quite important, 3 = very important	Distribution: ‘not really important’ (11.5 %), ‘quite important’ (50.5 %), ‘very important’ (38.0 %)
			Test-retest: κ = 0.64
Food as reward for good behavior (2 items)	How much do you agree or disagree with the following: ‘I offer sweets (e.g. lollies, ice cream, cake, pastries, sweet biscuits) to my child as a reward for good behavior’, ‘I offer my child his/her favorite foods in exchange for good behavior’ [28]	5 point: 1 = strongly disagree, 5 = strongly agree; range: 2-10	Mean: 4.3 ± 1.8; α = 0.78
			Test-retest: κ = 0.50-0.55
Sedentary behaviors as reward for good behavior (2 items)	How much do you agree or disagree with the following: ‘I let my child watch TV as a reward for good behavior’, ‘I let my child play computer/video games in exchange for good behavior’ [28]	5 point: 1 = strongly disagree, 5 = strongly agree; range: 2-10	Mean: 4.3 ± 2.0; α = 0.86
			Test-retest: κ = 0.46-0.55
Rules to limit sedentary behaviors (5 items)	‘My child is not allowed to watch TV/play PlayStation©/Nintendo© until his/her homework is done’, ‘During meal times, I do not allow the TV to be on’, ‘My child must be supervised when watching TV’, ‘My child must be supervised on the Internet or when playing PlayStation©/Nintendo©’, ‘I limit the amount of time my child spends watching TV/using the computer (internet and games)’	5 point: 1 = strongly disagree, 5 = strongly agree; range 5-25	Mean: 17.9 ± 3.3; α = 0.69
			Test–retest: items 1–4, ICC = 0 · 77-0 · 90; item 5, κ = 0.51
Feelings about food enjoyment (1 item)	How much do you agree or disagree with the following: ‘It gives me pleasure to give my children food they enjoy’ [28]	5 point: 1 = strongly disagree, 5 = strongly agree; range: 1-5	Mean: 3.9 ± 0.8
			Test-retest: κ = 0.47
Beliefs about food enjoyment (1 item)	How much do you agree or disagree with the following: ‘I believe in letting children enjoy food treats/rewards’ [28]	5 point: 1 = strongly disagree, 5 = strongly agree; range: 1-5	Mean: 3.4 ± 0.9
			Test-retest: κ = 0.48
Home access to physical activity equipment (11 items)	Which of the following do you have outside of your home or in your yard? ‘swimming pool/spa’, ‘trampoline’, ‘basketball ring’	0 = no, 1 = yes; range: 1-11	Mean: 7.8 ± 1.9
	Does your child have access to the following things at home? ‘balls’, ‘bats/racquets/golf clubs’, ‘bikes’, ‘home gym equipment’, ‘rollerblades’, ‘skateboard’, ‘skipping rope’, ‘scooter’		Test-retest: ≥ 89 % agreement [27]
Home access to equipment for sedentary behavior (6 items)	Does your child have access to the following things at home? ‘free to air TV’, ‘pay TV’, ‘video/DVD player’, ‘PlayStation©/Nintendo©/Gameboy©/X-box©’, ‘computer’, ‘internet’	0 = no, 1 = yes; range: 0-6	Mean: 4.1 ± 1.2
			Test-retest: ≥ 91 % agreement [27]
Child has a television in their bedroom (1 item)	Does your child have a TV in his/her bedroom?	0 = no, 1 = yes	Distribution: ‘no’ (84.5 %), ‘yes’ (15.5 %)
			Test-retest: ≥ 91 % agreement [29]
*Social environment*			
Social norms for physical activity (3 items)	How much do you agree or disagree with the following: ‘Lots of kids we know play sport’, ‘Lots of kids we know walk or cycle to school’, ‘Lots of kids we know play outdoors’	5 point: 1 = strongly disagree, 5 = strongly agree; range: 3-15	Mean: 11.6 ± 1.9; α = 0.57
			Test-retest: 62-73 % agreement
Social norms for unhealthy eating (2 items)	How much do you agree or disagree with the following: ‘Lots of kids we know eat fast food often’, ‘Lots of kids we know drink soft drink often’	5 point: 1 = strongly disagree, 5 = strongly agree; range: 2-10	Mean: 6.6 ± 1.9; α = 0.80
			Test-retest: κ = 0.53-0.57
Social norms for eating fruit (1 item)	How much do you agree or disagree with the following: ‘At my child’s school, lots of kids eat fruit often’	5 point: 1 = strongly disagree, 5 = strongly agree; range: 1-5	Mean: 3.9 ± 0.8
			Test-retest: κ = 0.51
*Neighborhood environment (subjective)*			
Mothers’ perception of neighborhood physical activity environment (1 item)	How much do you agree or disagree with the following: ‘The neighborhood I live in has lots of good places for my child to play and be active’ [30]	5 point: 1 = strongly disagree, 5 = strongly agree; range: 1-5	Mean: 3.7 ± 1.0
			Test-retest: κ = 0.54 [30]
Neighborhood child-friendliness/knowledge/liking (2 items)	How much do you agree or disagree with the following? ‘My child knows our local area very well’, ‘My child likes living in our local area’	5 point: 1 = strongly disagree, 5 = strongly agree; range: 2-10	Mean: 8.3 ± 1.4; α = 0.60
			Test-retest: 73-80 % agreement
Neighborhood social network (3 items)	How much do you agree or disagree with the following? ‘My child often visits other children and families in my area’, ‘My child’s friends live too far away from home to see on a regular basis’, ‘There are not many other children nearby for my child to play or hang around with’	5 point: 1 = strongly disagree, 5 = strongly agree (the latter 2 items were reverse coded); range: 3-15	Mean: 11.2 ± 2.6; α = 0.76
			Test-retest: 58-58 % agreement
Neighborhood personal safety (4 items)	How much do you agree or disagree with the following statements about your local neighborhood? ‘My neighborhood is safe for children’, ‘My neighborhood is safe for my child to walk/cycle around in the daytime’, ‘My child would be safe walking home from a bus or train stop’, ‘Concerns about stranger danger prevent my child from going outside in my local area’	5 point: 1 = strongly disagree, 5 = strongly agree (the latter item was reverse coded); range: 4-20	Mean: 13.5 ± 3.2; α = 0.80
			Test-retest: 51-77 % agreement
Neighborhood road safety (4 items)	How much do you agree or disagree with the following statements about your local neighborhood? ‘There are major barriers to walking/cycling that make it hard for my child to get from place to place (e.g. freeways, major roads)’, ‘There are no lights/crossings/pedestrian overpasses for my child to use’, ‘My child would have to cross several roads to get to areas where he/she can play or hang out’, ‘My child would have to cross a busy road/major highway to get to areas where he/she can play or hang out’	5 point: 1 = strongly disagree, 5 = strongly agree (all items reverse coded); range: 4-20	Mean: 12.4 ± 3.7; α = 0.79
			Test-retest: κ = 0.53-0.59
Neighborhood availability and quality of healthy foods (3 items)	How much do you agree or disagree with the following? ‘A large selection of fruit and vegetables are available in my neighborhood’, ‘The fresh fruit and vegetables in my neighborhood are of high quality’, ‘A large selection of low-fat products are available in my neighborhood’	5 point: 1 = strongly disagree, 5 = strongly agree; range: 3-15	Mean: 11.4 ± 2.4; α = 0.83
			Test-retest: κ = 0.65-0.71
*Neighborhood environment (objective) – derived from Geographic Information System data*			
Availability of a fast food outlet	Fast food outlet within 2000 m walking distance from participant’s home	0 = none, 1 = one or more	Distribution: ‘none’ (61.0 %), ‘one or more’ (39.0 %)
Availability of a supermarket	Supermarket within 2000 m walking distance from participant’s home	0 = none, 1 = one or more	Distribution: ‘none’ (26.0 %), ‘one or more’ (74.0 %)
Availability of a green grocer	Green grocer within 2000 m walking distance from participant’s home	0 = none, 1 = one or more	Distribution: ‘none’ (49.0 %), ‘one or more’ (51.0 %)
Availability of a swimming pool	Swimming pool within 2000 m walking distance from participant’s home	0 = none, 1 = one or more	Distribution: ‘none’ (48.0 %), ‘one or more’ (52.0 %)
Availability of a playground	Playground within 800 m walking distance from participant’s home	0 = none, 1 = one or more	Distribution: ‘none’ (43.0 %), ‘one or more’ (57.0 %)

#### Social environment

Measures of social norms related to children’s physical activity and eating behaviors were reported by mothers. These have been described in Table 1 and included perceptions of: social norms for physical activity; social norms for unhealthy eating; and social norms for eating fruit.

#### Neighborhood environment

Measures of the neighborhood environment were reported by mothers (Table 1). These included measures of perceptions of: the neighborhood physical activity environment; neighborhood familiarity; neighborhood social network; neighborhood personal safety; neighborhood road safety; and neighborhood availability and quality of healthy foods. Additionally, objective measures of the neighborhood environment were assessed using geographic information system (GIS) data. Data on the spatial location of various neighborhood amenities were collated from multiple sources including online directories, commercially available spatial datasets, and (state and local) government spatial datasets. The geographic information system ArcGIS (version 9.2) was used to calculate the number of fast food outlets, supermarkets, greengrocers (fresh produce market), and public swimming pools within a 2000 m walking distance of the child’s home, and the number of playgrounds within an 800 m walking distance. Whilst we acknowledge that the choice of buffer size may potentially impact on the findings [19], the buffer sizes used in this study represent conceptually appropriate distances to nearby facilities to which parents might drive (2000 m) or children might walk or cycle (800 m)[20]. Due to the uneven distributions of these measures, all objective environment variables were treated as dichotomous, and categorized as either the child having none, or one or more, of each amenity within 2000 m (800 m for playgrounds) of their home.

### Statistical analyses

Descriptive statistics (means, proportions) were used to characterize the sample. Separate linear regression models was used to examine associations between each of the individual, social and environmental factors reported at T1 and zBMI at T2, adjusting for baseline (T1) zBMI. Each predictor that was significantly (*p* <0.05) associated with zBMI at T2 was included in a fully adjusted multivariable model.

Similarly, logistic regression models were was used to examine associations between the individual, social and environmental factors reported at T1 and resilience to unhealthy weight gain, with the reference group being those children classified as ‘Not resilient to unhealthy weight gain’. Each predictor significantly (*p* <0.05) associated with resilience to unhealthy weight gain was included in a fully adjusted multivariable model (where applicable). All statistical analyses were conducted in Stata version 12 (StataCorp, TX) and included adjustment of the standard errors for clustering by area (the unit of recruitment). Child age and gender, maternal education, and area of residence (urban/rural) were considered as potential confounders. To be included as a confounder, variables had to demonstrate a statistically significant association with at least one of the outcomes (zBMI or resilience to unhealthy weight gain) and at least one of the environmental exposures. Only maternal education met this criteria and was thus included as a covariate in all regression models.

## Results

Sociodemographic characteristics of the sample are shown in Table 2. Children were on average 9 years old and mothers were on average 39.1 years old at baseline. Around a third of mothers had high levels of education. Mean zBMI was significantly lower at baseline (T1) but was higher at follow-up (T2) among children classified as not resilient to weight gain. Change in zBMI between T1 and T2 was significantly greater among those classified as not resilient to weight gain.

**Table 2:** Sociodemographic characteristics of children

Characteristic	All children		Resilient to unhealthy weight gain^b^		Not resilient to unhealthy weight gain^b^		*p* ^a^
	(*n* = 200)		(*n* = 133)		(*n* = 67)		
	n	Mean or %	n	Mean or %	n	Mean or %	
T1 Child age: Mean (SD)	200	9.0 (2.2)	133	8.9 (2.2)	67	9.2 (2.4)	.272
Child gender (%)							
Male	92	46.0	66	49.6	26	38.8	.147
Female	108	54.0	67	50.4	41	61.2	
T1 Maternal age: Mean (SD)	200	39.1 (4.6)	133	38.9 (4.7)	67	39.4 (4.6)	.525
T1 Maternal education (%)							
Low	40	20.0	27	20.3	13	19.4	.404
Medium	92	46.0	57	42.9	35	52.2	
High	68	34.0	49	36.8	19	28.4	
Area of residence							
Urban	66	33.0	42	31.6	24	35.8	.547
Rural	134	67.0	91	68.4	43	64.2	
T1 Child zBMI: Mean (SD)	200	−0.1 (0.7)	133	0.0 (0.7)	67	−0.2 (0.7)	.042
T2 Child zBMI: Mean (SD)	200	0.0 (0.8)	133	−0.2 (0.7)	67	0.4 (0.8)	<.0005
Change in zBMI between T1 and T2: Mean (SD)	200	0.0 (0.5)	133	−0.3 (0.3)	67	0.6 (0.4)	<.0005

^a^ Significance level for comparison between resilient and non-resilient children

^b^ ’Resilient to unhealthy weight gain’ defined as children who were healthy weight at baseline (T1) and whose change in zBMI between T1 and T2 was classified as small or medium; ‘Not resilient to unhealthy weight gain’ defined as children who were healthy weight at baseline (T1) and whose change in zBMI between T1 and T2 was classified as large

Only one factor, maternal efficacy for preventing their child from engaging in sedentary behaviors (B = −0.03, 95 % CI: −0.06, 0.00), was significantly associated with T2 BMI z-score (Table 3). This relationship was such that greater maternal efficacy regarding sedentary behaviors at T1 was associated with lower zBMI at T2. Having rules to limit sedentary behaviors (OR = 1.14, 95 % CI: 1.03, 1.25) was a significant predictor of being resilient to unhealthy weight gain (Table 4). None of the other variables were significantly associated with resilience.

**Table 3:** Associations from linear regression analysis between home, social and neighborhood environmental factors (reported at T1) and T2 zBMI (n = 200)†

Home, social and neighborhood environment factors	B	(95 % CI)	*p*
*Home environment*			
Maternal efficacy for child doing physical activity	0.00	(−0.02, 0.01)	.888
Maternal self-efficacy for preventing child from engaging in sedentary behaviors	−0.03*	(−0.06, 0.00)	.026
Maternal self-efficacy for child eating healthily	0.00	(−0.02, 0.01)	.621
Parental social support for physical activity	0.00	(−0.01, 0.02)	.523
Importance of doing physical activity as a family			
Not really important (reference category)			
Quite important	−0.14	(−0.44, 0.15)	.333
Very important	−0.13	(−0.43, 0.17)	.394
Food as reward for good behavior	−0.02	(−0.07, 0.02)	.309
Sedentary behaviors as reward for good behavior	−0.01	(−0.05, 0.02)	.519
Rules to limit sedentary behaviors	−0.02	(−0.04, 0.00)	.127
“It gives me pleasure to give my children food they enjoy”	0.00	(−0.07, 0.08)	.964
“I believe in letting children enjoy food treats/rewards”	0.03	(−0.07, 0.13)	.512
Home access to physical activity equipment	−0.01	(−0.04, 0.03)	.756
Home access to equipment for sedentary behaviors	0.06	(−0.01, 0.13)	.078
Child has a television in their bedroom	0.07	(−0.14, 0.27)	.503
*Social environment*			
Social norms for physical activity	0.00	(−0.04, 0.04)	.984
Social norms for unhealthy eating	0.02	(−0.02, 0.07)	.296
Social norms for eating fruit	−0.04	(−0.13, 0.04)	.282
*Neighborhood environment (subjective)*			
Neighborhood physical activity environment	−0.02	(−0.07, 0.03)	.411
Neighborhood familiarity	0.03	(−0.01, 0.08)	.151
Neighborhood social network	−0.01	(−0.04, 0.01)	.254
Neighborhood personal safety	−0.01	(−0.03, 0.02)	.581
Neighborhood road safety	0.01	(−0.01, 0.02)	.557
Neighborhood availability and quality of healthy foods	0.02	(−0.01, 0.05)	.281
*Neighborhood environment (objective)*			
Fast food outlet within 2000 m walking distance	0.09	(−0.07, 0.24)	.278
Supermarket within 2000 m walking distance	0.03	(−0.12, 0.18)	.710
Green grocer within 2000 m walking distance	−0.06	(−0.21, 0.10)	.471
Swimming pool within 2000 m walking distance	−0.13	(−0.27, 0.01)	.074
Playground within 800 m walking distance	0.08	(−0.05, 0.21)	.207

* *p* <0.05

^†^ Adjusted for child’s baseline zBMI, maternal education, and clustering by suburb

**Table 4:** Associations from logistic regression analysis between home, social and neighborhood environmental factors (reported at T1) and resilience to unhealthy weight gain (n = 200)†

Home, social and neighborhood environment factors	OR	(95 % CI)	*p*
*Home environment*			
Maternal efficacy for child doing physical activity	1.00	(0.94, 1.06)	.898
Maternal self-efficacy for preventing child from engaging in sedentary behaviors	1.12	(0.99, 1.27)	.063
Maternal self-efficacy for child eating healthily	1.00	(0.94, 1.07)	.887
Parental social support for physical activity	0.97	(0.93, 1.02)	.219
Importance of doing physical activity as a family			
Not really important (reference category)			
Quite important	1.06	(0.45, 2.53)	.890
Very important	1.08	(0.45, 2.60)	.867
Food as reward for good behavior	1.06	(0.88, 1.27)	.537
Sedentary behaviors as reward for good behavior	1.05	(0.91, 1.21)	.501
Rules to limit sedentary behaviors	1.14*	(1.03, 1.25)	.013
“It gives me pleasure to give my children food they enjoy”	0.97	(0.70, 1.35)	.858
“I believe in letting children enjoy food treats/rewards”	0.82	(0.54, 1.23)	.335
Home access to physical activity equipment	1.03	(0.89, 1.18)	.717
Home access to equipment for sedentary behaviors	0.84	(0.65, 1.08)	.171
Child has a television in their bedroom	0.67	(0.34, 1.33)	.253
*Social environment*			
Social norms for physical activity	0.94	(0.82, 1.09)	.412
Social norms for unhealthy eating	0.93	(0.76, 1.13)	.457
Social norms for eating fruit	1.14	(0.80, 1.61)	.461
*Neighborhood environment (subjective)*			
Neighborhood physical activity environment	1.14	(0.86, 1.51)	.354
Neighborhood familiarity	0.84	(0.69, 1.02)	.077
Neighborhood social network	1.04	(0.94, 1.14)	.454
Neighborhood personal safety	1.02	(0.94, 1.11)	.588
Neighborhood road safety	1.03	(0.95, 1.12)	.520
Neighborhood availability and quality of healthy foods	0.96	(0.86, 1.08)	.493
*Neighborhood environment (objective)*			
Fast food outlet within 2000 m walking distance	0.76	(0.38, 1.50)	.424
Supermarket within 2000 m walking distance	0.98	(0.57, 1.69)	.951
Green grocer within 2000 m walking distance	1.34	(0.76, 2.38)	.313
Swimming pool within 2000 m walking distance	1.58	(1.00, 2.49)	.051
Playground within 800 m walking distance	0.87	(0.52, 1.48)	.618

Odds ratios (ORs) greater than one indicate higher odds of being resilient to unhealthy weight gain

* *p* <0.05

^†^ Adjusted for maternal education and clustering by suburb

## Discussion

This study sought to examine predictors of resilience to unhealthy weight gain among children living in socioeconomically disadvantaged neighborhoods. It was hypothesized that a range of home, social and neighborhood environment factors would play a role in predicting increases in zBMI and resilience to unhealthy weight gain among children. Although a large number of potential predictors were examined, only two were found to be significantly associated with zBMI or resilience to unhealthy weight gain. Mother’s efficacy for preventing their child engaging in sedentary behavior was associated with smaller increases in zBMI over time, while having rules to prevent sedentary behavior was associated with resilience to unhealthy weight gain over time. None of the social or neighborhood environment measures were associated with zBMI or resilience to unhealthy weight gain. This suggests that programs aimed at supporting parents to limit children’s sedentary behavior may be particularly important in preventing unhealthy weight gain in socioeconomically disadvantaged families.

There is relatively little literature with which to compare these findings, with the two most relevant studies being the EAT [21] and CLAN [22] studies. In the EAT study, involving almost 2800 US adolescents in grades 6–12, Larson et al. [21] examined an extensive range of potential contextual influences on obesity. They found that a number of characteristics of the home, family and peer environments were consistently associated with higher BMI z-scores in boys and girls, with some neighborhood environment characteristics also being associated with BMI z-score among girls. While the EAT study examined a comprehensive range of correlates, it was based on a cross-sectional design, and was focused on identifying the correlates of unhealthy weight gain rather than resilience to unhealthy weight gain. The CLAN study involved a five year follow up of 300 Australian children aged 10–12 years. That study showed that having unmarried parents, maternal physical activity role modeling and the number of home sedentary items were positively associated with BMI *z*-score among boys. Among girls, having unmarried parents and maternal sedentary role modeling were positively associated, and number of sedentary behavior rules and physical activity items were inversely associated with BMI *z*-score among girls. Despite CLAN being longitudinal in design and including objective and subjective measures capturing different aspects of the local neighborhood, like EAT it was not focused on understanding the predictors of resilience to unhealthy weight gain. It is nonetheless noteworthy that like the current study, neighborhood environmental characteristics were not significant predictors of adiposity in CLAN. In addition, another US study also reported that a child’s residential food environment was not associated with obesity risk [23]. These results are not inconsistent with those reported in the adult literature. For example, a recent review of evidence on associations of the physical environment and weight status among adults concluded that, with few exceptions, the available research does not provide robust evidence that the physical environment influences adult weight status [24].

There are several possible study limitations that could also explain why so few significant associations were found, and so the findings should be interpreted with caution. It may be that we assessed the wrong variables and/or measured them poorly. This seems unlikely however, since the measures we used were based on those identified from the literature and on well-established theoretical models. Nonetheless, other unmeasured factors such as children’s school environment and preferred mode of transport between home and school may be important and worth including in future studies. It could also be that that within the socioeconomically disadvantaged neighborhoods that we sampled there was little variability in the predictor variables. However, an examination of the distribution of these variables suggested this was not the case (see Table 1). The length of exposure (three years) to the predictor variables may not have been adequate to have influenced the children’s weight, or there could have been changes in the predictor variables over the follow-up period that were not accounted for. It could also be that our definition of resilience to unhealthy weight gain, which was data-dependent and included those children in the bottom two tertiles of zBMI increase, captured both ‘healthy’ and ‘less healthy’ children. However, even when we examined zBMI continuously we found that few of the exposure variables were predictive. Finally, it may be that the small sample was underpowered to detect small associations.

## Conclusion

Despite these potential limitations, this study was novel and has strengths in its concurrent examination of a range of contextual influences in the home, social and neighborhood environments, in its use of objectives measures of the neighborhood environment, in employing a prospective study design. It is also unique in focusing on children living in socioeconomically disadvantaged neighborhoods, and in seeking to examine resilience to unhealthy weight gain. The findings suggest that strategies to support parents to limit their children’s sedentary behavior may be required to prevent unhealthy weight gain in families living in socioeconomically disadvantaged communities.
